# ‘Balloon screen’ technique: a case report of a novel bailout strategy for accurate stent deployment in severely calcified coronary lesions complicated by dissection

**DOI:** 10.1093/ehjcr/ytag118

**Published:** 2026-02-13

**Authors:** Masataka Yoshinaga, Takashi Muramatsu, Kenya Nasu, Akane Miyazaki, Eiichi Watanabe

**Affiliations:** Department of Cardiology, Fujita Health University Bantane Hospital, 3-6-10 Otobashi, Nakagawa-ku, Nagoya, Aichi 454-8509, Japan; Department of Cardiology, Fujita Health University Hospital, 1-98 Dengakugakubo, Kutsukake-cho, Toyoake, Aichi 470-1192, Japan; Department of Cardiology, Mie Heart Center, 2227-1 Meiwacho, Takigun, Mie 515-0302, Japan; Department of Cardiology, Fujita Health University Bantane Hospital, 3-6-10 Otobashi, Nakagawa-ku, Nagoya, Aichi 454-8509, Japan; Department of Cardiology, Fujita Health University Bantane Hospital, 3-6-10 Otobashi, Nakagawa-ku, Nagoya, Aichi 454-8509, Japan

**Keywords:** Balloon screen, Bailout technique, Calcified plaque, Coronary dissection, Rotational atherectomy, Case report

## Abstract

**Background:**

Severely calcified coronary lesions may require atherectomy and imaging guidance, yet procedural dissection can create a false lumen that repeatedly captures devices, risking inadvertent false-lumen stenting.

**Case summary:**

A 77-year-old man with Canadian Cardiovascular Society class III angina had severe diffuse calcification in the left anterior descending artery (ACC/AHA type B2; SYNTAX I 22; SYNTAX II 48.7). Although anatomical complexity was moderate, the SYNTAX II score was driven by advanced age and lower extremity artery disease. After rotational atherectomy (1.5-mm burr) and high-pressure scoring balloon angioplasty, guidewire exchange was complicated by migration into a false lumen behind circumferential calcium. Although a second wire was advanced into the true lumen using a double-lumen catheter, repeated attempts to deliver a drug-eluting stent deviated into the false channel. A semi-compliant balloon was therefore advanced into the false lumen and gently inflated to temporarily block it (‘balloon screen’), enabling stent delivery over the true-lumen wire. An Onyx Frontier 3.0 × 38 mm stent was deployed, and intravascular ultrasound confirmed excellent expansion. The patient was discharged the next day and remained asymptomatic at 6-month follow-up; key steps are shown in Supplementary material online, *Videos S1*–S3.

**Discussion:**

The balloon screen technique provides a practical bailout when stent delivery repeatedly tracks a false lumen after dissection in heavily calcified lesions. Creating a temporary physical barrier within the false channel, it promotes accurate true-lumen stenting, but careful balloon handling is required.

Learning points

- The balloon screen technique is an effective bailout strategy when stent delivery tracks a false lumen in dissected, heavily calcified coronary lesions.
- The balloon screen technique can reduce the risk of false-lumen stenting and related complications in complex percutaneous coronary interventions.

## Introduction

Heavily calcified coronary lesions are associated with lower percutaneous coronary intervention (PCI) success rates and a higher risk of complications, including incomplete stent expansion, malapposition, edge dissection, and coronary perforation.^1^ Contemporary strategies recommend lesion preparation with atherectomy and/or specialty balloons under intravascular ultrasound imaging (IVUS, OCT) guidance to optimize outcomes.^2,3^ While adequate lesion preparation improves stent expansion and outcomes, balloon angioplasty alone increases the risk of dissection or rupture.^4^ Although rare, the guidewire can sometimes enter a false lumen located behind the calcified plaque. If a stent is implanted into the false lumen, there is a high risk of complications, such as incomplete stent expansion, stent fracture, and side-branch occlusion.^5^ Here, we present a case where, despite successful volume reduction with RA, high-pressure cutting balloon angioplasty induced dissection and guidewire shift into a false lumen behind a calcified segment. While a second wire was advanced into the true lumen, the stent repeatedly migrated into the false lumen. By deploying a long semi-compliant balloon in the false channel, we performed a ‘balloon screen,’ which allowed precise stent deployment in the true lumen, successfully overcoming this rare and challenging scenario. To the best of our knowledge, this is the first report describing balloon screening of a false lumen to facilitate true lumen stenting in severe coronary calcification. In simple terms, this technique acts like a temporary roadblock in the wrong path, guiding the stent into the correct vessel.

## Summary figure

**Figure:**
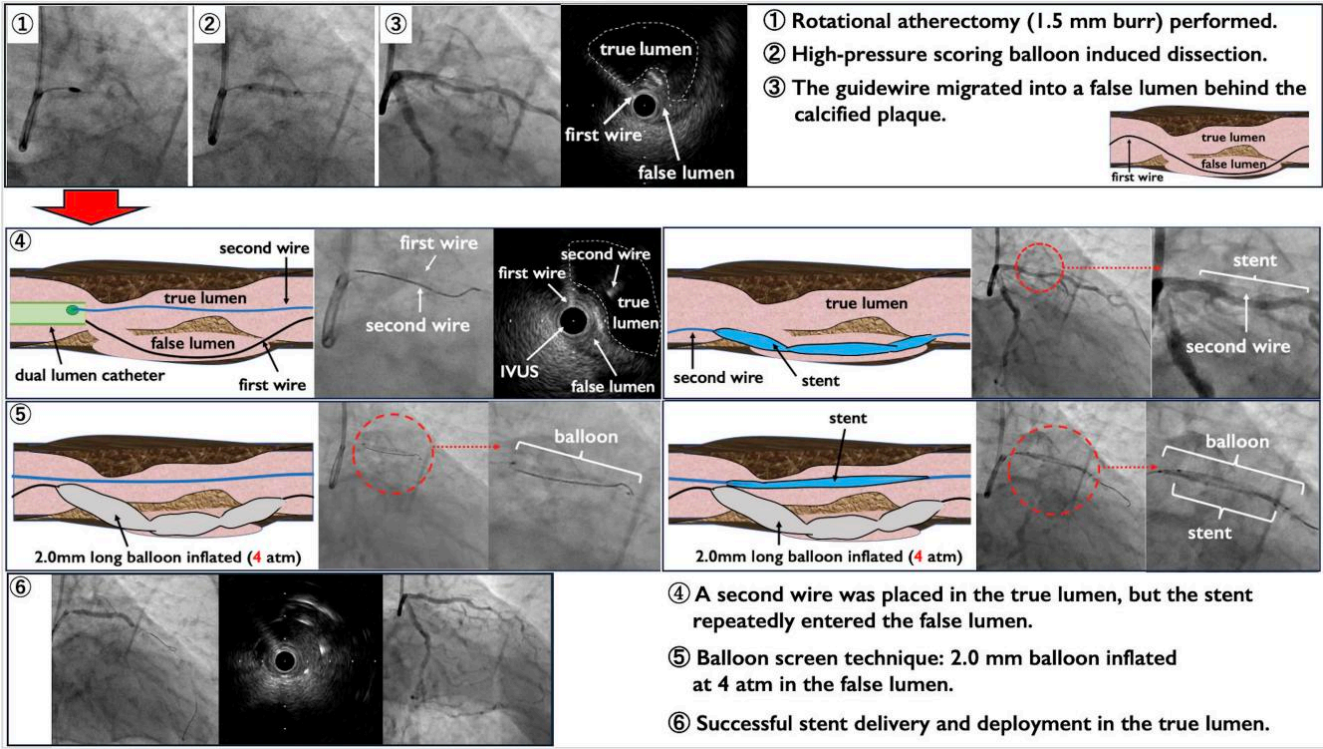
**Timeline of patient presentation, procedure and follow-up**: A graphical timeline summarizing the patient’s presentation, diagnostic work-up, intervention, and follow-up.

## Case presentation

**Figure 1:**
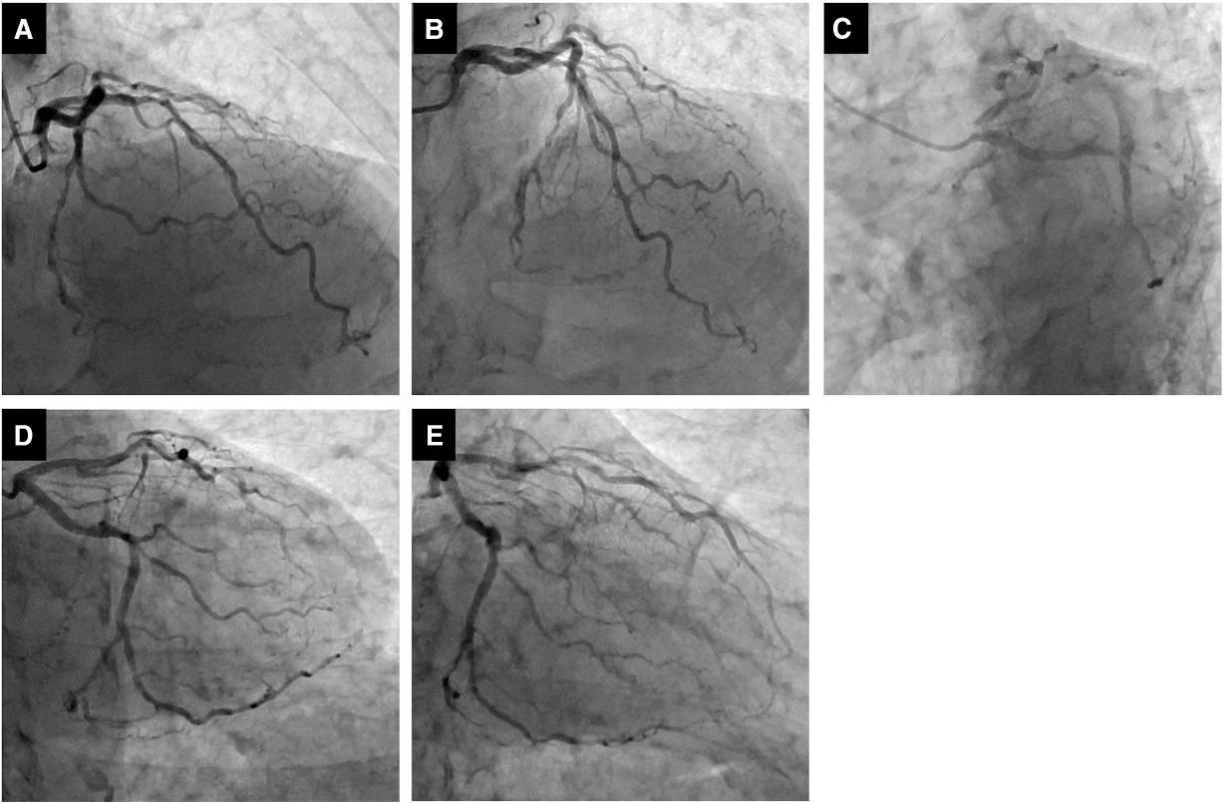
Previous procedure information. (*A*) Left coronary angiography (RAO 30°/CRA 30°). (*B*) Left coronary angiography (CRA 30°). (*C*) Left coronary angiography (LAO 30°/CAU 30°). (*D*) Left coronary angiography (CAU 30°). (*E*) Left coronary angiography (RAO 30°/CAU 30°).

**Figure 2:**
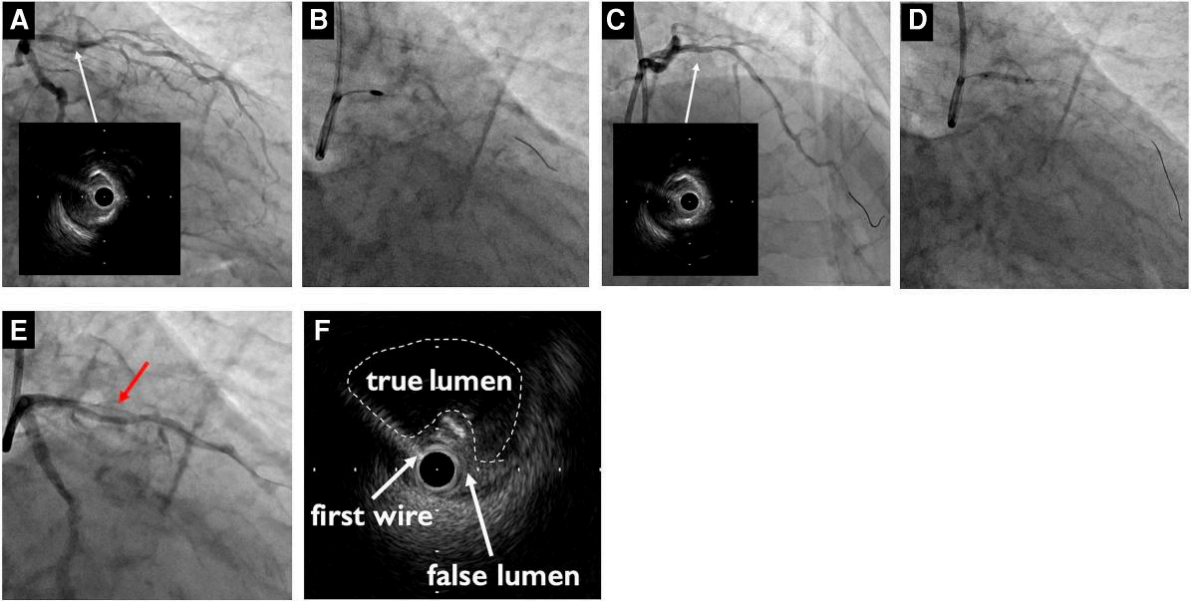
Percutaneous coronary intervention. (*A*) Angiography and intravascular ultrasound showing circumferential superficial calcification (>180° arc) (white arrow). (*B*) Rotational atherectomy performed with a 1.5 mm Rota Pro burr at 180 000 rpm. (*C*) Luminal gain achieved after atherectomy with no dissection (white arrow). (*D*) High-pressure scoring (cutting) balloon angioplasty. (*E*) The first guidewire migrated into a false lumen behind the dissected calcified segment (red arrow). (*F*) Intravascular ultrasound image obtained over the first guidewire located in the false lumen, demonstrating the spatial relationship between the true and false lumens.

**Figure 3:**
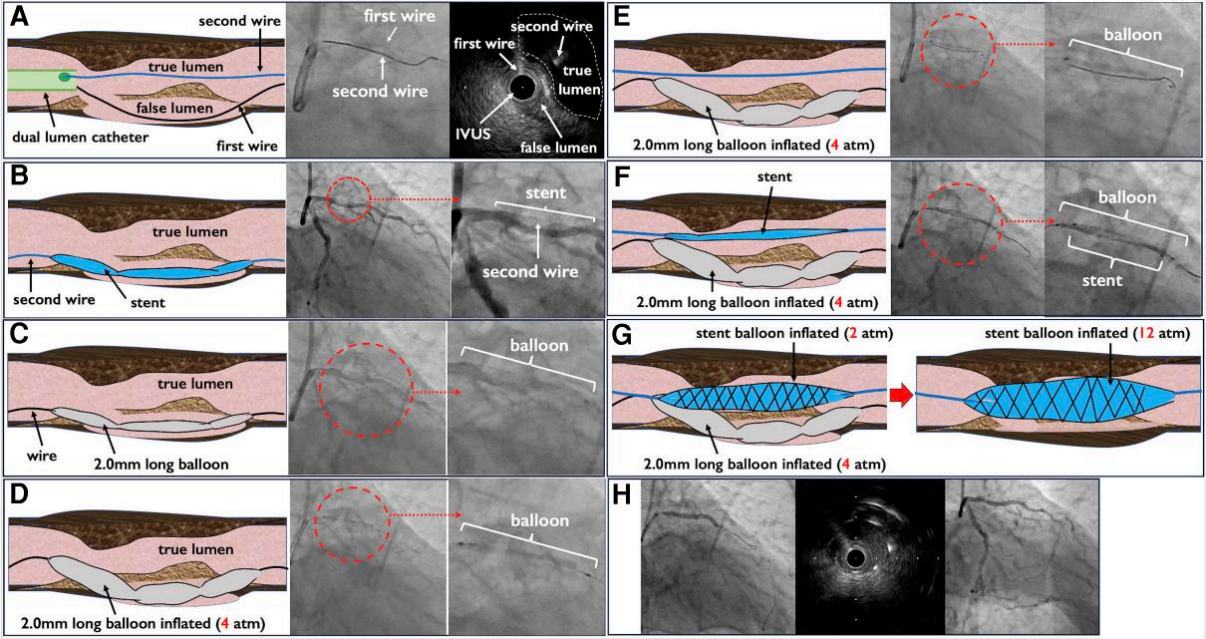
Balloon screen technique schema. (*A*) A second wire was advanced into the true lumen using a dual-lumen microcatheter. (*B*) Repeated stent delivery attempts resulted in migration into the false lumen behind the dissected segment. (*C*) A 2.0 × 30 mm semi-compliant balloon was advanced into the false lumen. (*D*) The balloon was gently inflated (4 atm) to screen the false lumen. (*E*) The stent was advanced over the true-lumen wire. (*F*) Slight inflation of the stent balloon (2 atm) allowed removal of the false-lumen balloon and wire. (*G*) The stent was then fully deployed to nominal pressure. (*H*) Final angiography and intravascular ultrasound confirmed successful stent deployment in the true lumen.

A 77-year-old man presented with CCS class III angina. He had hypertension. Resting electrocardiography showed no ischaemic changes, and echocardiography revealed preserved left-ventricular function (ejection fraction 62%). Fractional flow reserve angiography demonstrated values of 0.78 in the left anterior descending artery (LAD). Cardiac computed tomography and coronary angiography showed heavy calcified disease of the LAD lesion (ACC/AHA Type B2,^6^ SYNTAX I = 22; SYNTAX II = 48.7 with a predicted 4-year mortality of 28.7%) (*Figure 1A–1E*). SYNTAX II score was 48.7 owing to age and low creatinine clearance (CrCl) (49 mL/min) and history of lower-extremity artery disease (LEAD). A 7 F Super Backup 3.5 guiding catheter was introduced via the left radial artery (the right radial artery was small and had been used previously for peripheral interventions). A Sion Blue guidewire (ASAHI Intec, Aichi, Japan) crossed the LAD lesion, and AnteOwl IVUS (TERUMO, Tokyo, Japan) confirmed the extent of calcification. IVUS demonstrated circumferential superficial calcification over a 10 mm segment with a minimal luminal area of 2.0 mm^2^ (*Figure 2A*); the distal reference vessel diameter was 3.0 mm and the proximal reference diameter 3.5 mm. The Sion Blue wire was exchanged for a Rota Floppy wire (Boston Scientific, Natick, MA) using a microcatheter, and rotational atherectomy was performed with a 1.5 mm Rota Pro burr (Boston Scientific) at 180 000 rpm (*Figure 2B*). Two passes ablated superficial calcium and increased the luminal area (*Figure 2C*). After rotational atherectomy, the Rota wire was exchanged back to the workhorse wire using a trapping balloon. High-pressure scoring (cutting) KIZASHI 3.0 × 15 mm balloon angioplasty (KANEKA, Tokyo, Japan) was then performed to further modify the calcified plaque (*Figure 2D*). During subsequent manipulation, the guidewire migrated into a false lumen behind the dissected plaque (*Figure 2E*). The IVUS catheter was advanced over the first guidewire located in the false lumen to confirm wire position and vessel architecture. This wire was not suitable for stent delivery because it was located within the false lumen (*Figure 2F*). We adopted the following stepwise approach for each step. The procedural steps are demonstrated and a simple schema diagram is shown in *Figure 3A–3H*. A second Sion Blue wire was advanced into the true lumen using a dual-lumen microcatheter (*Figure 3A*). Four attempts to deliver a stent along this wire failed because the stent consistently entered the false channel (*Figure 3B*). We therefore advanced a ZINRAI 2.0 × 30 mm semi-compliant balloon (KANEKA) into the false lumen (*Figure 3C*) and inflated it gently to 4 atm to ‘screen’ the false lumen (*Figure 3D*). With the false lumen temporarily blocked, a guidewire was advanced into the true lumen. The stent was then delivered over this true-lumen wire and partially inflated at 2 atm (*Figure 3E* and *3F*). The false-lumen balloon and wire were then deflated and removed, and the stent balloon was inflated to nominal pressure (*Figure 3G*). An Onyx Frontier 3.0 × 38 mm drug-eluting stent (Medtronic, Minneapolis, MN) was selected considering its design characteristics, including a relatively thin strut platform and favourable deliverability, which were thought to be suitable for this complex lesion. IVUS confirmed good stent apposition and expansion with no edge dissection (*Figure 3H*). The procedure lasted 176 min; 210 mL of contrast and 2070 mGy of radiation were used. The patient recovered uneventfully, was discharged the following day and remained asymptomatic at 6-month follow-up. Key procedural steps are provided in Supplementary material online, *Videos S1*–S3. The clinical course and key procedural steps are summarized in *Table 1*, and the Summary figure highlights the Balloon Screen bailout step.

**Table 1:** Timeline of patient presentation, diagnostic work-up, procedural steps, and clinical outcomes

Time point	Key events and clinical details
Presentation	The patient presented with CCS class III angina and a history of lower extremity artery disease treated by endovascular therapy. Baseline investigations (ECG, echocardiography) were unremarkable. FFRangio values were 0.78 (LAD) and 0.86 (LCX).
Diagnostic work-up	Cardiac CT and coronary angiography revealed a heavy calcified LAD lesion (ACC/AHA Type B2, SYNTAX I = 22). SYNTAX II score was 48.7 owing to advanced age and low CrCl and history of LEAD. IVUS confirmed circumferential calcification (distal reference 3.0 mm; proximal reference 3.5 mm; and minimal luminal area 2.0 mm^2^).
Intervention	(1) Rotational atherectomy (1.5 mm burr) performed. (2) High-pressure scoring balloon-induced dissection. (3) The guidewire migrated into a false lumen behind the calcified plaque. (4) A second wire was placed in the true lumen, but the stent repeatedly entered the false lumen. (5) Balloon screen technique: 2.0 mm balloon inflated at 4 atm in the false lumen. (6) Successful stent delivery and deployment in the true lumen.
Post-procedure	Recovery: Uneventful; discharged on Day 1.
Follow-up	6-month follow-up: asymptomatic (no MACE).

Abbreviations: ACC/AHA, American College of Cardiology/American Heart Association; CCS, Canadian Cardiovascular Society; CT, computed tomography; ECG, electrocardiogram; FFRangio, angiography-derived fractional flow reserve; IVUS, intravascular ultrasound; LAD, left anterior descending artery; LCX, left circumflex artery; MACE, major adverse cardiovascular events.

## Discussion

In the present case, the cutting balloon may have contributed to the propagation of a dissection plane because superficial circumferential calcium can limit vessel compliance and concentrate mechanical stress at the edges of the cutting elements, facilitating intimal tearing and extension of an intramural haematoma. Adequate lesion preparation is essential for successful PCI in heavily calcified coronary arteries. Rotational atherectomy, scoring or cutting balloons, and intravascular imaging are recommended for plaque modification and procedural guidance.^2,7^ However, balloon inflation following atherectomy can occasionally cause dissection and guidewire entry into a false lumen. Stent implantation into a false lumen may lead to incomplete expansion, fracture, perforation, and side-branch occlusion. When a guidewire migrates into a false lumen despite careful lesion preparation, several bailout strategies—such as the parallel wire technique—can be considered.^5,8^ In our case these strategies failed, and a long semi-compliant balloon was used to temporarily occlude the false lumen, allowing the stent to be delivered along the true-lumen wire. This ‘balloon screen’ technique has not been widely described. Matsumura and colleagues recently reported a similar approach to facilitate true-lumen re-entry after intravascular lithotripsy–induced dissection, which underscores the potential utility of balloon screening in various scenarios.^9^ The balloon screen technique is conceptually simple and uses readily available balloons. Nevertheless, it carries risks: balloon entrapment or rupture within the false lumen, propagation of dissection/intramural haematoma, vessel perforation, difficulty advancing the balloon across tortuous anatomy, and potential compromise of side branches. Inflation pressure and duration should be minimized, and intravascular imaging should be used to confirm true-lumen wiring and adequate stent expansion. Because safe implementation often requires intravascular imaging and (in some cases) a dual-lumen microcatheter, availability and cost may limit use in some centres. The technique should be considered only after conventional bailout methods have failed. Larger series and longer follow-up are required to assess its safety and generalizability.

## Conclusion

The balloon screen technique is a simple, effective bailout strategy for ensuring accurate true-lumen stent deployment in dissected, heavily calcified coronary lesions when guidewires repeatedly enter a false lumen. Intravascular imaging and careful management of the false-lumen balloon are essential to minimize complications. Awareness of this method may assist interventional cardiologists facing similar complex scenarios.

## Lead author biography


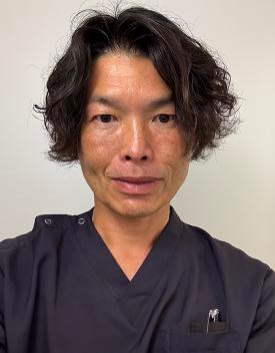


Masataka Yoshinaga was born in 1978 in Fukuoka, Japan. He graduated from Fujita Health University in 2006 and is currently serving as a lecturer at Fujita Health University Bantane Hospital in Aichi, Japan. His main areas of expertise and interest are percutaneous coronary intervention (PCI) and endovascular treatment (EVT).

## Supplementary Material

ytag118_Supplementary_Data

## Data Availability

All data generated or analysed during this study are included in this published article and its supplementary information files. Owing to privacy considerations, the raw patient data cannot be shared publicly but may be made available to qualified investigators upon reasonable request to the corresponding author.
