# Supplementary material for: ‘Balloon screen’ technique: a case report of a novel bailout strategy for accurate stent deployment in severely calcified coronary lesions complicated by dissection
Source: Eur Heart J Case Rep. 2026 Feb 13;10(3):ytag118. doi: 10.1093/ehjcr/ytag118 (PMC12959534; doi:10.1093/ehjcr/ytag118)
Supplement: ytag118_Supplementary_Data [file ytag118_supplementary_data.zip › Supplementary material.docx]

**Supplementary material**

Supplementary material is available at European Heart Journal – Case Reports online.

**Supplementary Video 1**. Repeated guidewire and stent migration into the false lumen behind the dissected, heavily calcified segment.

**Supplementary Video 2**. Balloon Screen technique: low-pressure balloon inflation within the false lumen to guide wire passage and stent delivery into the true lumen.

A 2.0-mm balloon was gently inflated at low pressure within the false lumen, temporarily blocking it and facilitating advancement of the guidewire and subsequent stent delivery into the true lumen.

**Supplementary Video 3**. Final angiographic result demonstrating satisfactory coronary flow without apparent complications, such as side-branch occlusion.
